# Recurrent Thyroid Storm Caused by a Complete Hydatidiform Mole in a Perimenopausal Woman

**DOI:** 10.1155/2020/8842987

**Published:** 2020-12-23

**Authors:** Anuradha Jayasuriya, Dimuthu Muthukuda, Preethi Dissanayake, Shyama Subasinghe

**Affiliations:** ^1^Diabetes and Endocrinology Unit, Colombo South Teaching Hospital, Kalubowila, Colombo, Sri Lanka; ^2^Diabetes and Endocrinology Unit, Sri Jayewardenepura General Hospital, Nugegoda, Colombo, Sri Lanka; ^3^Sri Jayewardenepura General Hospital, Nugegoda, Colombo, Sri Lanka

## Abstract

**Background:**

Gestational trophoblastic disease (GTD) which includes hydatidiform mole, invasive mole, placental site trophoblastic tumor, and choriocarcinoma is a rare cause of hyperthyroidism due to excess production of placental human chorionic gonadotrophin hormone (hCG) by tumor cells. Molecular mimicry between hCG and thyroid stimulating hormone (TSH) leads to continuous stimulation of TSH receptor by extremely high levels of hCG seen in these tumors. Consequently, biochemical and clinical hyperthyroidism ensues and it is potentially complicated by thyrotoxic crisis which is fatal unless urgent therapeutic steps are undertaken. *Case Description*. We present a 49-year-old perimenopausal woman who presented with recurrent thyroid storm and high output cardiac failure. The initial workup revealed suppressed TSH, high-free thyroxine (FT4), and free triiodothyronine (FT3) levels with increased vascularity of the normal-sized thyroid on ultrasonography. She was managed with parenteral beta blockers, steroids, and high-dose carbimazole. Her lower abdominal tenderness led to further investigations which revealed tremendously elevated beta-hCG and a snow storm appearance on transabdominal ultrasound suggestive of GTD. She underwent curative surgery and was diagnosed with complete hydatidiform mole postoperatively by histology.

**Conclusion:**

Recurrent thyroid crisis in gestational trophoblastic disease is an exceedingly rare presentation and that is highly fatal. This case highlights the importance of early detection and treatment of the etiology of thyrotoxicosis to eliminate mortality.

## 1. Introduction

The hormone hCG comprises of an *α*-subunit and a *β*-subunit. The former is structurally similar to the *α*-subunit of luteinizing hormone (LH), follicle-stimulating hormone (FSH), and thyroid-stimulating hormone (TSH) [1]. This similarity in TSH and hCG accounts for the continuous stimulation of TSH receptors in GTD where excessive production of hCG by trophoblastic tumor cells leads to hyperthyroidism.

Epidemiological studies have reported a broad variation in the incidence of GTD including hydatidiform mole (H mole) and choriocarcinoma. Japan and South East Asia have the highest reported incidence of H mole that has been estimated up to be 2 cases in 1000 pregnancies [2].

Molar pregnancy in postmenopausal women is a rare occurrence [3]. To date, there are only a small number of case studies and case series published in the previous literature about the gestational trophoblastic disease and hyperthyroidism [4, 5]. Even rarer are the case reports of thyroid storm and benign trophoblastic disease in perimenopausal women [6]. To our knowledge, our case represents the first description in the world literature of a complete hydatidiform mole (CHM) in a perimenopausal woman presenting with recurrent thyroid storm.

## 2. Case Report

A 49-year-old mother of 2 children presented with progressively worsening shortness of breath and orthopnea for 3 days. She had been experiencing intermittent fever, anorexia, nausea, and abdominal pain for 1 month, and she had developed sweating, tremors, and palpitations suggestive of hyperthyroidism. Her menstrual history was remarkable for a period of amenorrhoea for 3 months.

On examination, she was dyspneic with bilateral pedal edema. Her blood pressure was 180/110 mmHg, and jugular venous pressure was elevated. There were bibasal crepitations, and abdominal examination revealed a pelvic mass of a 14-week-sized uterus.

**Figure 1:**
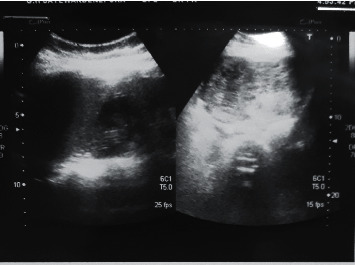
Ultrasound scan of the abdomen showing the typical snow storm appearance of the hydatidiform mole.

**Figure 2:**
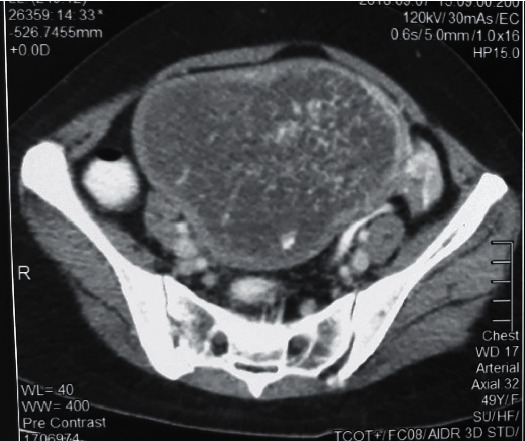
Contrast-enhanced CT appearance of the hydatidiform mole.

Basic investigations including full blood count and renal and liver function tests were normal. Chest X-ray showed diffuse infiltrates with bilateral small pleural effusions. 2D echo findings were compatible with high output cardiac failure. Thyroid functions revealed evidence of thyrotoxicosis with suppressed TSH of 0.009 mIU/mL, elevated free T3 of 10.89 pg/mL, and free T4 of 6.91 ng/dL. There was evidence of hypervascularity in thyroid ultrasonography. She had hyperthermia, atrial fibrillation, severe heart failure, and central nervous system disturbances fulfilling criteria for thyrotoxic crisis [7]. She was urgently commenced on high-dose carbimazole, parenteral beta blockers, and steroids. Because of persistent abdominal pain, ultrasound abdomen was performed which revealed the snow storm appearance that is a unique feature of gestational trophoblastic disease (Figure 1). Her serum beta-hCG level was substantially increased up to 146,092,800 mIU/mL, and contrast CT of abdomen confirmed a uterine mass measuring 12.6 cm × 8.9 cm × 9.0 cm (Figure 2).

Because of extremely high levels of hCG, the working diagnosis was established as choriocarcinoma and, therefore, she was prepared to be sent for chemotherapy at cancer institute of Sri Lanka. While awaiting chemotherapy, she developed the second episode of thyrotoxic crisis. At this point, her beta-hCG titer was 254,532,200 mIU/mL, TSH was0.022 mIU/mL, FT3 was 6.15 pg/mL, and FT4 was 2.89 ng/dL, and she received urgent parenteral furosemide, digoxin, hydrocortisone, and high-dose propylthiouracil.

**Figure 3:**
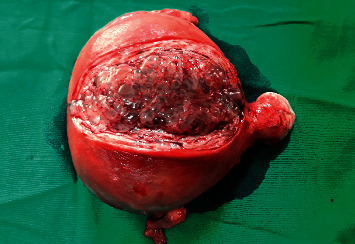
Specimen of the uterus showing the hydatidiform mole along the incision line of myometrium, a large mass of grape-like structures.

Preoperative optimization was carried out with antihypertensives (losartan, prazosin, indapamide, and propranolol), antipyretics, and adequate hydration. Cholestyramine and Lugol's iodine were added for further control of her hyperthyroid state. Following stabilization of her thyrotoxic crisis, a transabdominal hysterectomy and bilateral oophorectomy under general anesthesia was performed successfully under intraoperative hydrocortisone and esmolol infusions. Macroscopic appearance of the tumor was compatible with a hydatidiform mole (Figure 3).

The patient was managed by the multidisciplinary team involving the physician, endocrinologist, obstetrician, cardiologist, cardioelectrophysiologist, and anesthesiologist. The tumor was later confirmed by histology as a complete hydatidiform mole without evidence of myometrial invasion.

**Figure 4:**
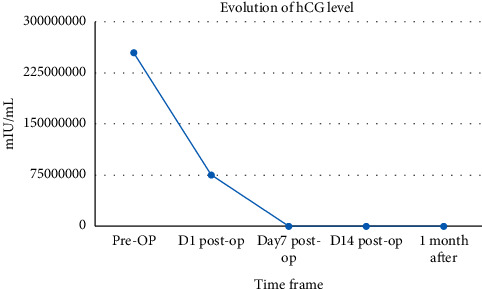
Evolution of beta-hCG level over the course of treatment.

Following surgery, she became clinically euthyroid and her thyroid functions and hCG level gradually improved over 3 months (Table 1) (Figure 4).

## 3. Discussion

The usual clinical trial of GTD includes uterine enlargement inconsistent with the period of amenorrhea, hyperemesis gravidarum, and markedly elevated serum hCG level. This patient presented with a period of amenorrhoea, abdominal pain, and irregular vaginal bleeding without vomiting in spite of very high levels of serum beta hCG. Occurrence of symptomatic hyperthyroidism in GTD is not common [7]. On review of the 196 patients with gestational trophoblastic neoplasia treated with chemotherapy in Sheffield since 2005, 14 (7%) had biochemical hyperthyroidism. Of these, only four had evidence of clinical hyperthyroidism [8]. GTD presenting with thyrotoxic crisis in perimenopausal women is extremely rare and restricted to case reports [9]. Although recurrent thyroid storm has been reported in association with thyroid cancer, there were no published literature on GTD presenting with recurrent thyroid crisis [10]. 

Early recognition of this clinical entity is important due to the complications associated with the hyperthyroid state mostly during surgery. Our patient had hyperthyroidism for 1-month duration, and she developed thyrotoxic crisis complicated with atrial fibrillation and heart failure while awaiting surgery. There has been only few reported cases of atrial fibrillation caused by hyperthyroidism secondary to hydatidiform moles [11]. The use of iodinated substances is a recognized trigger of thyrotoxic crisis (the Jod -Basedow phenomenon) and is reported in association with GTD [12, 13]. Use of iodinated contrast during imaging has possibly contributed to the development of recurrent thyrotoxic crisis in this patient. Because of urgent management with parenteral bata blockers and antithyroid drugs, her perioperative period was uncomplicated. The initial suspicion of choriocarcinoma in this patient can be explained by the fact that extremely high levels of serum hCG correlates better with an underlying choriocarcinoma rather than with an H mole [14].

Serum FT3 to FT4 ratio (FT3/FT4) is a useful tool for the differentiation of the cause of thyrotoxicosis. FT3/FT4 ratio of > 4.4 (10^−2^ pg/ng) strongly supports the diagnosis of Graves' disease, and a lower FT3/FT4 ratio of < 2.73 (10^−2^ pg/ng)) suggests other etiologies for hyperthyroidism [15]. It is a recognized fact that the conversion of T4 to T3 could be impaired in severe thyroid storm [16]. Japanese thyroid association conducted a nationwide survey which revealed that FT3 levels and the FT3/FT4 ratio were inversely proportional to the disease severity in thyroid storm [17]. In both episodes of thyrotoxic crisis, FT3/FT4 < 2.73 (10^−2^ pg/ng) was compatible with the above findings. In the fist episode, higher FT4 and FT3 levels suggest untreated severe hyperthyroidism and FT3/FT4 ratio < 2 (10^−2^ pg/ng) indicate underlying low T3 syndrome associated with euthyroid sick syndrome [18, 19]. During the second episode, comparatively lower FT3 and FT4 concentrations can be caused by antithyroid drug (ATD) therapy. Therefore the difference in the FT3/FT4 ratios at the both episodes of thyroid storm can be explained by multiple factors such as the underlying cause, the precipitant and the severity of thyroid crisis, commencement of ATDs, and non thyroidal illness syndrome. 

The definitive management of hydatidiform mole is either surgical evacuation of the molar tissue by hysterectomy or curettage if the patient is willing for further pregnancy. Despite the fact that surgery is curative, it can provoke thyroid crisis which is fatal. Hence perioperative management should be carried out with extreme precautions. There are few reported cases of surgical intervention for trophoblastic disease complicated with thyroid storm [20, 21]. The safety and effectiveness of prophylactic chemotherapy for gestational trophoblastic disease was assessed by several studies, and although prophylactic chemotherapy may reduce the progression of GTD with high malignancy risk, it is not currently recommended because of the adverse effect profile and concerns about drug resistance [22]. 

This patient underwent hysterectomy and oophorectomy because of the rare possibility of her GTD being malignant. However, it was confirmed by histology to be a benign complete H mole without invasion of surrounding tissues. She was followed up by monitoring of serial hCG levels weekly until negative and then monthly for 6 months.

## 4. Conclusion

Even though rare, GTD should be considered as an etiology for thyrotoxicosis in women of perimenopausal age. In fact, it is a diagnostic challenge to the clinician, and early recognition and preoperative stabilization with medical management is of paramount importance prior to curative surgery. Complete resection of these tumors leads to dramatic reduction of perioperative morbidity and mortality.

## Figures and Tables

**Table 1:** Evolution of thyroid function.

	Preoperative day		Postoperative day		
	1st episode of thyroid storm	2nd episode of thyroid storm	Day 5	Day 14	Day 60
TSH (mIU/L) (0.27–4.20 mIU/L)	0.009	0.022	0.012	1.38	0.562
Free T4 (ng/dL) (0.93–1.70 ng/dL)	6.91	2.89	1.69	2.64	1.270
Free T3 **(**pg/mL) (2.3–4.2 pg/mL)	10.89	6.15	1.77	1.17	
FT3/FT4 ratio (10^−2^ pg/ng)	1.576	2.128			

Analytical sensitivity for TSH: 0.008 mIU/mL, FT4: 0.1 ng/dL, and FT3: 0.2 pg/mL. Measured by using a fully automated immunoassay analyzer ADVIA centaur XP.

## Data Availability

The clinical data used to support the findings of this article are obtained from the bed head tickets stored in the record room of the Sri Jayewardenepura General Hospital, Nugegoda, Sri lanka. Other original reports including the clinic records and the diagnosis cards are with the patient.
